# Mucin utilization by gut microbiota: recent advances on characterization of key enzymes

**DOI:** 10.1042/EBC20220121

**Published:** 2023-04-18

**Authors:** Grete Raba, Ana S. Luis

**Affiliations:** 1 Department of Chemistry and Biotechnology, Tallinn University of Technology, Akadeemia tee 15 12618, Tallinn, Estonia; 2 Department of Medical Biochemistry and Cell Biology, University of Gothenburg, Box 440, 405 30 Gothenburg, Sweden

**Keywords:** enzyme activity, glycoside hydrolases, microbiology, mucins, sulfatases

## Abstract

The gut microbiota interacts with the host through the mucus that covers and protects the gastrointestinal epithelium. The main component of the mucus are mucins, glycoproteins decorated with hundreds of different *O*-glycans. Some microbiota members can utilize mucin *O*-glycans as carbons source. To degrade these host glycans the bacteria express multiple carbohydrate-active enzymes (CAZymes) such as glycoside hydrolases, sulfatases and esterases which are active on specific linkages. The studies of these enzymes in an *in vivo* context have started to reveal their importance in mucin utilization and gut colonization. It is now clear that bacteria evolved multiple specific CAZymes to overcome the diversity of linkages found in *O*-glycans. Additionally, changes in mucin degradation by gut microbiota have been associated with diseases like obesity, diabetes, irritable bowel disease and colorectal cancer. Thereby understanding how CAZymes from different bacteria work to degrade mucins is of critical importance to develop new treatments and diagnostics for these increasingly prevalent health problems. This mini-review covers the recent advances in biochemical characterization of mucin *O*-glycan-degrading CAZymes and how they are connected to human health.

## Introduction

**Figure 1:**
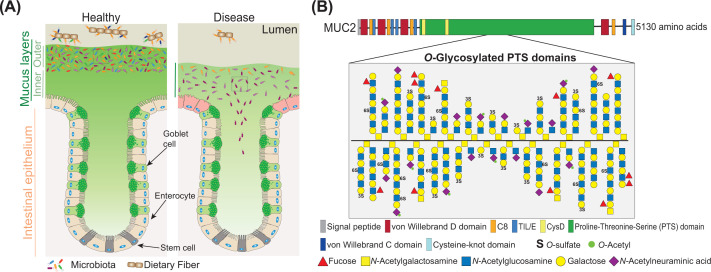
Overview of colonic mucus layers (**A**) In the healthy colon the mucus is organized as the inner and outer mucus layer, which have low and high bacterial colonization, respectively. The major components of the mucus layer are mucins that are synthesized and secreted by goblet cells. In diseases, such as ulcerative colitis, there is an increase in the mucus layer penetrability and bacteria can be found close to the inflamed intestinal epithelium. ( **B**) Schematic representation of the MUCIN 2 (MUC2). In the mucus layer, MUC2 is organized in a complex network due to N- and C-terminal disulfide bounds. This glycoprotein is heavily *O*-glycosylated in proline, threonine and serine (PTS) domains. The MUC2 monomer backbone has a molecular weight of around 500 kDa which goes up to 2.5 MDa after glycosylation.

The human gut harbours a complex and diverse microbial community, the gut microbiota, that has an essential role on human health. The gut microbiota colonizes the mucus layer that covers the intestinal epithelium [1]. The properties of this mucus layer are variable along the gastrointestinal (GI) tract. In the small intestine the mucus forms a single loose layer. However, in the colon the mucus is organized into two layers: the inner layer, attached to the epithelium, is tightly arranged and it is almost devoid of bacteria, whereas the outer mucus layer is loosely arranged and is heavily colonized by the microbiota (Figure 1A) [1]. This double-layer organization of the colonic mucus is essential to the host health. The outer layer provides the ideal habitat for the bacteria to thrive in the gut environment, while the impenetrable inner mucus layer acts as a barrier keeping the bacteria away from the intestinal epithelium preventing close contact and inflammation [2]. The major components of the mucus are mucins, heavily *O*-glycosylated glycoproteins (Figure 1B). These host glycans have a major role in determining which bacteria can successfully colonise the host [1,3,4]. At the same time, some members of the human microbiota have been shown to be able to forage on mucins and if excessive, the bacterial degradation of mucins can lead to alterations on microbiota composition and a thinner and penetrable mucus barrier (Figure 1A) [5–7]. It is now clear that alterations on both the microbiota and the mucus barrier function, can lead to multiple diseases such as obesity, diabetes, colorectal cancer and inflammatory bowel disease (IBD) [6–9]. Therefore, understanding the mechanisms of degradation and utilization of mucins by the gut microbiota could have a key role on finding treatments to prevent or control diseases characterized by the excessive bacterial erosion of the mucus layer.

## Select members of the microbiota can degrade mucin O-glycans

**Figure 2:**
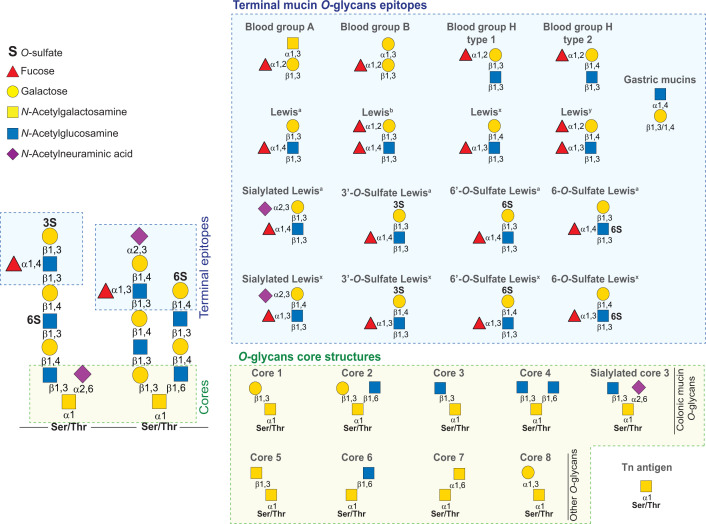
Schematic representation of mucin *O*-glycan structures Different terminal epitopes and cores structures highlighted inside the blue and yellow boxes, respectively. Sugars are shown according to the Symbol Nomenclature for Glycan system [60].

Mucins can be membrane-bound or secreted. Membrane mucins are a part of the glycocalyx of mucosal surfaces where they are involved in cell signalling and act as a last defence line to keep bacteria away from the epithelium [10]. Secreted mucins are the main components of the mucus and give it its viscous gel-like properties. The mucus composition along the GI tract is variable with Mucin5AC (MUC5AC) and Mucin2 (MUC2) being the most common mucins in the stomach and intestine, respectively (Figure 1B) [1]. The mucin protein backbone is characterized by having multiple repeating units of proline-threonine-serine (PTS) domains that are extensively decorated with complex *O*-glycans made of *N*-acetylgalactosamine (GalNAc), galactose (Gal) and *N*-acetylglucosamine (GlcNAc) chains capped with sialic acid, sulfate and fucose (Fuc) in multiple positions and blood groups generating multiple terminal epitopes (Figure 2). A single mucin can contain more than a hundred different *O*-glycan structures with these sugars making up to 80% of the total mucin molecular weight [1,11]. Importantly, the *O*-glycosylation varies spatially along the GI tract and between different species. In humans and pigs, there is an increasing gradient of sialyation and sulfation along the Gl tract, whereas the opposite is observed for fucosylation [12–16]. Such variations in glycosylation will have a significant impact on the microbiota utilization of these complex glycans. Indeed, a recent study showed that *Akkermansia muciniphila*, a bacterium known to grow on *O*-glycans of porcine gastric mucins (PGM), failed to grow on colonic *O*-glycans [17]. Therefore, to address the mechanisms of degradation of mucins, it is critical to understand the structural variability of mucin *O*-glycosylation.

To degrade *O*-glycans, the gut bacteria encode multiple carbohydrate active enzymes (CAZymes) such as glycoside hydrolases (GHs), carbohydrate esterases (CE) and sulfatases. CAZymes are classified into families based of sequence similarity in the databases CAZy (GHs and CEs) and SulfAtlas (only sulfatases) [18,19]. Inside a family the proteins share a conserved fold, catalytic mechanism and active site residues. Additionally, depending on the cleavage site, the GHs and sulfatases are also classified as either exo- or endo-active with the former hydrolysing glycans from one end and the latter within the glycan chain [20].

## CAZymes active on terminal mucin capping structures

The substitution of mucins with sialic acid, sulfate, fucose and blood groups creates a barrier against degradation by the microbiota (Figure 2). To utilize mucin glycans the bacteria need to encode specific enzymes that remove the various capping structures. The different enzymes active on the mucin substitutions are described below.

### Sulfatases

**Figure 3:**
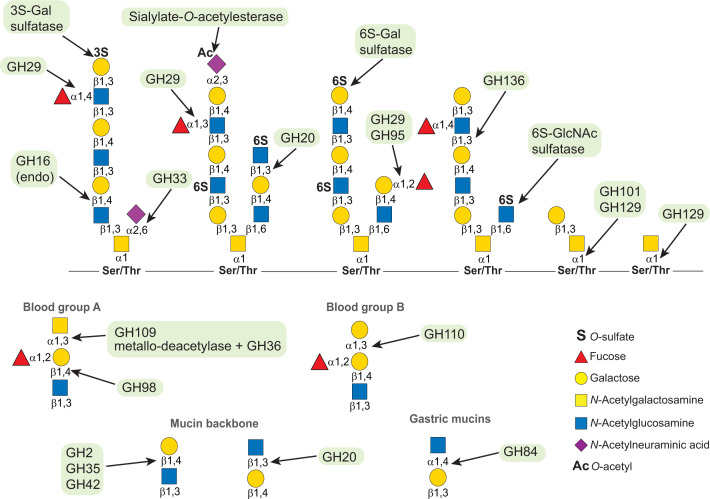
Specificity of mucin *O*-glycans CAZymes The arrows point to the linkage cleaved by the different enzymes shown inside green boxes. Sugars are shown according to the Symbol Nomenclature for Glycan system [60]; GHXXX, glycoside hydrolase family XXX.

In mucins, sulfation occurs *O*-linked to Gal at positions 3, 4 and 6 (3S-, 4S- and 6S-Gal, respectively) and to GlcNAc in position 6 (6S-GlcNac). Sulfation on Gal is always terminal whereas 6S-GlcNAc can be terminal or internal (Figure 2) [17]. Sulfation is variable between species with 3S-Gal and 6S-GlcNAc being common in porcine colonic mucins while human colonic mucins also show a high level of 6S-Gal [13,15,21]. The sulfation on 4S-Gal is present on saliva mucins that transit trough the GI tract where they become an available carbon source for the gut microbiota [22]. Several members of the human microbiota encode sulfatases that remove the sulfate capping group allowing access to *O*-glycan (Figure 3) [17]. The microbial sulfatase activity has been previously associated with colitis, a type of IBD [23]. Additionally, it has been shown that *Bacteroides thetaiotaomicron*, a prominent member of the human microbiota, can lead to colitis in a susceptible animal model and the development of this disease is dependent of active sulfatases [24]. Despite the link between sulfatases and disease, these enzymes remain poorly characterized. Recently, a study identified the activity of 12 of the 28 sulfatases encoded by *B. thetaiotaomicron* [17]. Surprisingly, 11 on these enzymes are active on sulfated linkages found on mucin *O*-glycans. Together these enzymes can potentially remove all the sulfate groups found in mucin. However, only 4 sulfatases were found active on colonic mucin *O*-glycans, cleaving specifically sulfation of 3S-Gal or 6S-GlcNAc. The present study also identified the first sulfatase active on 3S-linked to GalNAc (3S-GalNAc), a linkage not yet identified in mucin or other host glycans [17]. The structural characterization of *B. thetaiotaomicron* sulfatases showed for the first time that these enzymes evolved different specificity determinants to specifically recognize the target linkage within different glycan contexts [17,25]. Importantly, the present study identified a single 3S-Gal sulfatase (BT1636) as essential to *B. thetaiotaomicron* growth on colonic *O*-glycans and *in vivo* fitness during a competitive gut colonization [25]. A similar key role of sultases in gut colonization was also shown in *Bacteroides fragilis*. BF3134, a homolog of the 6S-GlcNAc enzymes characterized in *B. thetaiotaomicron*, has been shown to have a key role in *B. fragilis in vivo* fitness in presence of an invading strain suggesting that sulfatases are also important in securing colonization within the gut [26].

Sulfation in 6S-GlcNAc is a major substitution found in colonic mucins of human, pigs and mice. To remove this sulfation some gut bacteria, such as *Prevotella* and *Bifidobacterium bifidum*, developed an alternative mechanism of overcoming the 6S-GlcNAc blockage. These bacteria do not use sulfatases but instead their genomes encode a GH20 that cleaves terminal 6S-GlcNAc (Figure 3) [27,28]. In *B. bifidum*, this 6-sulfo-β-*N*-acetylglucosaminidase is expressed at the bacteria cell surface and it has been shown to be active on PGM [28]. However, the specificity of these GH20 enzymes against colonic mucins remains unclear. Overall, all the mucin active enzymes that cleave sulfation have evolved to recognize sulfate groups at the terminal positions suggesting that these enzymes act in the initial stages of mucin degradation by microbiota members.

### Sialidases

On *O*-glycans, terminal sialic acid residues are found α2,3/2,6-linked to Gal and GalNAc (Figure 2) [13]. Humans only have *N*-acetylneuraminic acid (Neu5Ac) while pigs also have *N*-glycolylneuraminic acid [17]. Colonic mucins are highly sialylated and the ability to cleave and/or utilize sialic acid has a key role in gut colonization. To cleave sialic acid most bacteria encode one or several GH33 sialidases (Figure 3) [29]. These enzymes can be classified either as trans-sialidases that cleave α2,3 linkages, or hydrolytic sialidases that cleave all sialic acid linkages. Trans-sialidases were identified on *Ruminococcus gnavus* where the activity of these enzymes converts Neu5Ac into 2,7-anhydro-Neu5Ac that is specifically metabolized by this bacterium [30,31]. This modification of the sialic acid confers an advantage within the gut environment where *R. gnavus* does not need to compete with other bacteria to utilize this *O*-glycan monosaccharide. Recently, it was shown that the deletion of the trans-sialidase impaired the *R. gnavus* growth on mucin and, *in vivo*, the mutant had a fitness defect when competing with the WT strain [31]. Interestingly, in monocolonized mice the mutant strain lost the ability to colonize the colonic mucus layer closer to the intestinal epithelium indicating that the trans-sialidase is required to colonize the niches within the mucus barrier [31]. The bacteria relying on hydrolytic sialidases that utilize sialic acid have all the required enzymes for Neu5Ac metabolism grouped together into Nan clusters [29]. For bacteria with incomplete Nan clusters, Neu5Ac can be used in interspecies crosstalk. For example, *B. thetaiotaomicron* can release Neu5Ac from mucin glycans, but lacks the enzymes for its further metabolism, thereby leaving the Neu5Ac residues to be consumed by other bacteria in the environment such as *Salmonella typhimurium* and *Clostridium difficile* who lack sialidase-activity [32]. More recently, founding member of the GH156 family displayed activity against α2,3/2,6-Neu5Ac on short oligosaccharides [33]. This new enzyme was initially isolated from hot spring metagenomes but the taxonomic display shows that the genome of gut microbiota members, such as, *Parabacteroides merdae* and *Parabacteroides johnsonii*, also encode GH156 enzymes [18]. Although the characterized enzyme failed to show activity in glycans presented on the surface of cancer cells, the activity of these enzymes was not yet addressed on complex *O*-glycans [34].

### Fucosidases

On mucin *O*-glycans, fucose can be α1,2-linked to Gal or α1,3/1,4-linked to GlcNAc which determines the different H type or Lewis antigen epitopes (Figure 2) [11]. To cleave fucose the gut bacteria rely on fucosidases from families GH29 and GH95 (Figure 3). While the GH95 are known to be α1,2-fucosidases, members of GH29 family can act on all fucose linkages found in mucins [18]. The GH29 enzymes can be divided into two subfamilies (A or B) according to their specificity. GH29-A have a broad specificity and act on *p*-nitrophenyl(*p*NP)-α-fucoside, while GH29-B specifically cleave α1,3/1,4-fucose [35]. In presence of mucin, bacteria up-regulate the expression of multiple fucosidases indicating that the removal of fucose is critical to growth and/or to access other sugars in *O*-glycans [3,6,36]. Additionally, fucose released by gut commensals, like *B. thetaiotaomicron*, can also be scavenged by pathogens, such as enterohaemorrhagic *Escherichia coli*, leading to the increased expression of virulence factors and gut colonization [37]. Despite the importance of fucosidases, the characterization of the substrate specificity of these enzymes remains limited to few members and such activities have only been shown against synthetic oligosaccharides and not complex *O*-glycans. *B. thetaiotaomicron* encodes nine GH29 and four GH95 genes that can be associated in mucin degradation. So far, only 4 of the GH29s enzymes have been characterized. BT2970 and BT4136 are active on *p*NP-α-fucoside [35,38]. BT1625 and BT2192 are 1,3/1,4-fucosidases. BT2192 specifically acts on trisaccharide substrates (Lewis^a^ and Lewis^x^) while BT1625 is active against all Lewis antigens [35,39]. For *B. fragilis* only four GH29s out of the 12 predicted fucosidases have been previously characterized [40]. BF0810 was only active on *p*NP-α-fucoside while BF0028 also showed weak activity against α1,2 and α1,3 linkages. BF3242 and BF3591 were able to cleave α1,2/1,3/1,4-fucose. However, BF3591 displays a preference to α1,3-Fuc found in Lewis^x/y^, common epitopes found in mucins [40]. Recently a study aiming the characterization of fucosidase activity in different *Ruminococcus gnavus* strains showed that two GH29s encoded by the E1 and ATCC 29149 strains preferably cleave α1,3/1,4-Fuc and α1,2-Fuc, respectively. Interestingly, only one enzyme from E1 strain (E1_10125) was able to accommodate terminal sialic acid modifications, a common feature in *O*-glycans [36].

An alternative mechanism to remove fucose can be cleaving the terminal oligosaccharides containing this substitution. Recently, a member of GH136, a family populated with lacto-*N*-biosidases, was shown to specifically release Lewis^a^ and Lewis^x^ from human milk oligosaccharides, a substrate that is similar to *O*-glycans [41]. This enzyme identified in *Roseburia inulinivorans* evolved to recognize Fuc-α1,4-GlcNac at the subsite-1 (Figure 3). Interestingly, it was shown that this bacterium also encodes a GH29 specific towards α1,4-fucose [41]. Although these enzymes were not shown to be active on mucin *O*-glycans it would be interesting to determine if mucin degrading bacteria encode GH136 or/and α1,4-specific GH29s that are able to cleave terminal fucosylated oligosaccharides.

### *Enzymes cleaving A/B blood groups* and other additional capping structures

To access mucins gut bacteria have also evolved to encode enzymes that remove blood groups. To remove blood group A and B bacteria can deploy α-*N*-acetylgalactosaminidases (GH109) and α-galactosidases (GH110), respectively (Figure 3) [42]. Very few members of these families have been characterized so far. In GH110 it has been shown that *B. thetaiotaomicron* and *B. fragilis* encode enzymes that specifically recognize the trisaccharide B group [Gal-α1,3(Fucα1,2-)Gal] and enzymes that are active on both linear and branched Gal-α1,3-Gal oligosaccharides [43]. Recently a GH109 enzyme of *A. muciniphila* was shown to be display both α-retaining and β-inverting mechanisms releasing the same product (α-GalNac) from α-GalNac and β-GalNAc substrates, respectively [44]. This dual mechanism can allow the bacterium to access different glycans such as mucins or glycolipids that cover epithelium cells. To remove A/B blood groups, bacteria can also encode endo-β1,4-galactosidases (GH98 enzymes) (Figure 3) [45]. Of relevance, a GH98 enzyme from *Ruminococcus gnavus* ATCC29149 was shown to release GalNAc-α1,3(Fucα1,2-)Gal from PGM [46]. Interestingly, this enzyme enables *R. gnavus* E1 (a strain lacking GH98s) to grow on the released trisaccharide and also the underlaying *O*-glycans exposed after enzyme treatment. It was suggested that in *Ruminococcus* species the GH98s can be critical to the utilization of mucin glycans [46]. Recently a study aiming to identify novel blood group cleaving enzymes encoded by the microbiota revealed an alternative mechanism to remove these epitopes. *Flavonifractor plautii* deploys two enzymes to specifically remove blood group A [47]. First, a metallo-GalNAc-deacetylase converts the terminal GalNAc to *N*-galactosamine (GalN) that acts as a substrate for a novel GH36 α-galactosaminidase (Figure 3) [47]. The sequential action of these enzymes allows the quick conversation of blood group A to group H demonstrating that the gut microbiota evolved different mechanisms to remove terminal epitopes found on mucins.

Additional terminal capping structures, such as α1,4-GlcNAc, can be found in gastric mucins [48]. Although this epitope is not found on colonic mucin, several gut bacteria encode α-*N*-acetylglucosaminidases (GH89) that can remove this linkage from mucins passing through the gut lumen (Figure 3) [6,11]. At last, *O*-acetylation in sialic acid is a common modification on colonic mucins that blocks the access of bacterial sialidases [13]. To overcome this barrier, some microbiota members express sialate-*O*-acetylesterases (Figure 3). Although not much is known about these enzymes, EstA, an acetylesterase expressed by *B. thetaiotaomicron* and *B. fragilis*, enables *E. coli* to access sialic acid and grow on mucin [49]. Interestingly, EstA is a specific 9-*O*-acetylesterase that requires the spontaneous migration of the *O*-acetyl group from carbon 7 to 9 to be active [49]. Since *O*-acetylation is a common feature of human colonic mucins, further studies are required to characterize novel *O*-glycan specific *O*-acetylesterases and understand the role of such enzymes in gut colonization.

## CAZymes active on mucin *O*-glycans backbone and cores

Upon the cleavage of the terminal capping sugars bacteria can access the protected *O*-glycans backbone. The cleavage of these can happen through the sequential action of β-galactosidases and β-*N*-acetylglucosaminidases that cleave β-Gal and β-GlcNAc. Several bacteria known as able to grow on *O*-glycans express several enzymes with such specificities; however, almost none of these enzymes have ever been tested on colonic mucins. Internal Gal is β1,4-linked to GlcNAc whereas terminal Gal can be linked β1,3 or β1,4 generating type 1 or type 2 epitopes, respectively. Additionally, core 1 and 2 also contain Gal-β1,3-GalNAc. (Figure 2) [11]. These linkages can be cleaved by β-galactosidases from families GH2, GH35 and GH42 (Figure 3) [18]. *A. muciniphila* genome encodes 6 GH2 enzymes. Only 3 of these proteins were characterized showing that this bacterium has evolved to target all the different linkages found in mucin [50]. Amuc_0771, Amuc_1666 and Amuc_0924 specifically cleave Gal-β1,3-GlcNAc, Gal-β1,4-GlcNAc and Gal-β1,3-GalNAc, respectively. Only Amuc_0771 and Amuc_0924 display activity on PGM which is consistent with the high abundance of type 1 linkages on this substrate [50]. A GH35 from *A. muciniphila* was also shown to be active on core 1 and core 2 structures. However, the activity of mucins was never shown for this enzyme [51]. After Gal has been cleaved, the β-*N*-acetylglucosaminidases (GH20 and GH84) will then cleave the exposed GlcNAc (Figure 3). These enzymes have been mostly characterized on short glycans or against *p*NP substrates [52–55]. Additionally, families GH101 and GH129 are populated with enzymes that share structural similarities and target mucin cores with different specificities (Figure 3) [56]. GH129 enzymes are highly abundant in *Bifidobacterium* and specifically cleave Tn antigen (GalNAc-α1-Ser) but can also act on core 1 (Gal-β1,3-GalNAc-α1-Ser) [57]. The family GH101 is encoded in genomes of Actinobacteria and several Firmicutes and these endo-enzymes specifically target core 1 [58].

Endo-active enzymes that cleave within *O*-glycans offer an additional mechanism to access these sugars without the need to remove capping groups. Such endo-acting *O*-glycanases were recently identified in GH16 family (Figure 3) [59]. Interestingly, these endo-β1,4-galactosidases require the removal of sialic acid prior to the activity [59]. Since these enzymes are encoded by commensals such as *B. thetaiotaomicron*, *B. fragilis* and *A. muciniphilia*, the blockage of this enzymatic activity by sialic acid can represent an adaptation to limit the access of these bacteria to mucin glycans within the colonic mucus layer.

## Summary

The gut microbiota encodes an immense repertoire of CAZymes that allow these bacteria to access different carbon sources. Some bacteria have evolved to utilize complex mucin *O*-glycans and it is known that the access to these host glycans can have a critical role in gut colonization. To overcome the linkage diversity found on *O*-glycans, mucin degrading bacteria express multiple CAZymes that must work in tandem to degrade these complex glycans. Many putative mucin-degrading enzymes have been predicted from the transcriptomic and proteomic data but the characterization of these enzymes remains a challenge. The lack of an accessible source of colonic mucins and the requirement of specialized equipment to analyze *O*-glycan samples containing hundreds of unique structures have limited the study of CAZymes on the substrate targeted by these enzymes. To work around such limitations, the specificity of *O*-glycans CAZymes have been mostly determined on short mucin-type oligosaccharides and it is now possible to start to puzzle together all the required enzymes to break down highly *O*-glycosylated mucins. However, the known characterized enzymes are encoded by different bacteria and the mechanism of *O*-glycans degradation by a single bacterium remains unclear. Additionally, in order to understand the mechanisms of mucin degradation by the microbiota it is essential to understand the complexity and the variations on glycosylation within these host glycans. Therefore, future studies should consider the characterization of CAZymes on colonic mucins in order to identify novel key enzymes required to initiate the bacterial degradation of *O*-glycans. Such enzymes will likely reveal new therapeutic targets that can be used to restore the microbiota community in inflammatory diseases, such as IBD. Overall, by increasing our understanding of how different gut microbiota species metabolize host glycans we will start to disclose the key microbiota-human mucin interaction that drive the gut colonization with a healthy microbiota.

## Summary

- Some gut microbiota members are able to break down and utilize complex mucin *O*-glycans that are found in the colonic mucus layer.
- Gut bacteria express specific enzymes to target all the different linkages found on *O*-glycans. However, most of the enzymes lack a characterization on complex colonic mucin *O*-glycans.
- Enzymes cleaving terminal epitopes, such as sulfatases and sialidases, have a key role in bacterial gut colonization.
- Understanding the mechanisms of mucin *O*-glycans degradation and utilization by microbiota members can reveal key enzymes that are potential drug targets in diseases associated with the increase of bacterial foraging on mucins and the disruption of the protective colonic mucus layers, such as IBD, obesity and colorectal cancer.

## Open Access

Open access for this article was enabled by the participation of University of Gothenburg in an all-inclusive *Read & Publish* agreement with Portland Press and the Biochemical Society.

## Abbreviations

CAZyme: carbohydrate-active enzyme
CE: carbohydrate esterase
Fuc: fucose
Gal: galactose
GalNAc: *N*-acetylgalactosamine
GH: glycoside hydrolase
GI: gastrointestinal
GlcNAc: *N*-acetylglucosamine
IBD: inflammatory bowel disease
Muc2: Mucin2
Neu5Ac: *N*-acetylneuraminic acid
PGM: porcine gastric mucin
*p*NP: *p*-nitrophenyl
PTS: proline-threonine-serine
